# *Nlrp12* mutation causes C57BL/6J strain-specific defect in neutrophil recruitment

**DOI:** 10.1038/ncomms13180

**Published:** 2016-10-25

**Authors:** Tyler K. Ulland, Nidhi Jain, Emma E. Hornick, Eric I. Elliott, Gwendolyn M. Clay, Jeffrey J. Sadler, Kathleen A. M. Mills, Ann M. Janowski, A. Paige Davis Volk, Kai Wang, Kevin L. Legge, Lokesh Gakhar, Mohammed Bourdi, Polly J. Ferguson, Mary E. Wilson, Suzanne L. Cassel, Fayyaz S. Sutterwala

**Affiliations:** 1 Inflammation Program, University of Iowa Carver College of Medicine, Iowa City, Iowa 52242, USA; 2 Interdisciplinary Program in Molecular and Cellular Biology, University of Iowa Carver College of Medicine, Iowa City, Iowa 52242, USA; 3 Department of Medicine, Cedars-Sinai Medical Center, Los Angeles, California 90048, USA; 4 Interdisciplinary Program in Immunology, University of Iowa Carver College of Medicine, Iowa City, Iowa 52242, USA; 5 Medical Scientist Training Program, University of Iowa Carver College of Medicine, Iowa City, Iowa 52242, USA; 6 Department of Pediatrics, University of Iowa Carver College of Medicine, Iowa City, Iowa 52242, USA; 7 Department of Biostatistics, University of Iowa College of Public Health, Iowa City, Iowa 52242, USA; 8 Department of Pathology, University of Iowa Carver College of Medicine, Iowa City, Iowa 52242, USA; 9 Department of Biochemistry, University of Iowa Carver College of Medicine, Iowa City, Iowa 52242, USA; 10 Protein Crystallography Facility, University of Iowa Carver College of Medicine, Iowa City, Iowa 52242, USA; 11 Molecular and Cellular Toxicology Section, Laboratory of Molecular Immunology, National Heart, Lung and Blood Institute, National Institutes of Health, Bethesda, Maryland 20892, USA; 12 Department of Internal Medicine, University of Iowa Carver College of Medicine, Iowa City, Iowa 52242, USA; 13 Veterans Affairs Medical Center, Iowa City, Iowa 52246, USA; 14 Department of Microbiology, University of Iowa Carver College of Medicine, Iowa City, Iowa 52242, USA

## Abstract

The inbred mouse strain C57BL/6J is widely used in models of immunological and infectious diseases. Here we show that C57BL/6J mice have a defect in neutrophil recruitment to a range of inflammatory stimuli compared with the related C57BL/6N substrain. This immune perturbation is associated with a missense mutation in *Nlrp12* in C57BL/6J mice. Both C57BL/6J and NLRP12-deficient mice have increased susceptibility to bacterial infection that correlates with defective neutrophil migration. C57BL/6J and NLRP12-deficient macrophages have impaired CXCL1 production and the neutrophil defect observed in C57BL/6J and NLRP12-deficient mice is rescued by restoration of macrophage NLRP12. These results demonstrate that C57BL/6J mice have a functional defect in NLRP12 and that macrophages require NLRP12 expression for effective recruitment of neutrophils to inflammatory sites.

## Abstract (web summary)

The role of NLRP12 in immunity to bacterial infection is controversial as varied and contrasting results have been published using C57BL/6 mice. Here the authors shed light on this issue, showing that unlike C57BL/6N mice, C57BL/6J mice have a missense point mutation in NLRP12 that is associated with defective neutrophil recruitment.

The genetic background of a transgenic or knockout mouse has an important, but often overlooked, role in its phenotype. The founder C57BL/6J substrain originated in the 1920s and has been maintained at the Jackson Laboratories since 1948. A colony was established at the National Institutes of Health in 1951 and designated as the C57BL/6N substrain. Over the intervening 65 years there has been substantial genetic drift. Comparison of the genome between these two substrains revealed 40 single nucleotide polymorphisms (SNPs) and 30 indels^1^,^2^,^3^. This genetic difference is relevant because large projects have utilized different substrains for their work. The International Mouse Phenotyping Consortium, for example, uses C57BL/6N embryonic stem cells for the generation of knockout mice. By contrast, the Mouse Genome Sequencing Consortium has relied on the C57BL/6J substrain. In addition, many commercially available knockout mice are on a mixed background with varying genetic contributions from both C57BL/6J and C57BL/6N substrains. Both substrains are widely used in biomedical research and hence it is critical to understand the phenotypic differences, their genetic causes, and in particular how these differences affect the outcomes of immunological studies using these mice.

The nucleotide-binding domain and leucine-rich repeat containing receptor (NLR) family of proteins is a group of intracellular receptors that are important for sensing both pathogen-associated and danger-associated molecular patterns^4^. NLRP12 was initially identified as a member of the NLR family reported to attenuate inflammatory responses^5^,^6^,^7^. NLRP12 has also been shown to inhibit colonic inflammation and tumorigenesis in mouse models through negative regulation of nuclear factor kappa-light-chain (NF-κB)^8^,^9^. The role for NLRP12 in controlling bacterial infections is less clear. Vladimer *et al*.^10^ suggest that NLRP12 serves a pro-inflammatory role during infection with a modified *Yersinia pestis* strain through its ability to form a caspase-1 activating inflammasome that regulates interleukin (IL)-1β and IL-18 secretion. In marked contrast, Zaki *et al*.^11^ found an anti-inflammatory role for NLRP12 showing that NLRP12-deficient mice were resistant to infection with *Salmonella typhimurium* due to increased NF-κB and extracellular signal-regulated kinases activation. In yet a separate study, Allen *et al*.^12^ identified no role for NLRP12 in the *in vivo* host response to *Klebsiella pneumoniae* and *Mycobacterium tuberculosis*. Given these markedly discrepant findings and postulated mechanisms of action of NLRP12 in bacterial infection, a closer examination is warranted.

In this study, we demonstrate that C57BL/6J mice have a defect in recruitment of neutrophils to inflammatory sites *in vivo*. This immune perturbation in C57BL/6J mice is associated with a missense mutation within the leucine-rich repeat domain of *Nlrp12* resulting in an arginine to lysine change (R1034K). The failure of neutrophil migration in C57BL/6J mice was phenocopied in *Nlrp12*^*−/−*^ mice. By comparing C57BL/6N, C57BL/6J and *Nlrp12*^*−/−*^ mice we further demonstrate that the role of NLRP12 in the recruitment of neutrophils to the site of inflammation is critical for host defense against bacterial pathogens. Expression of C57BL/6N NLRP12 in macrophages is required to mediate appropriate neutrophil recruitment. Furthermore, there is a defect in macrophages from C57BL/6J and *Nlrp12*^*−/−*^ mice to produce the neutrophil chemotactic chemokine CXCL1 in response to inflammatory stimuli. Altogether, our findings demonstrate that NLRP12 controls neutrophil migration to inflammatory sites and that a missense mutation in *Nlrp12* in C57BL/6J mice renders these mice with a functional NLRP12 deficit.

## Results

### Reduced neutrophil recruitment in C57BL/6J mice

**Figure 1:**
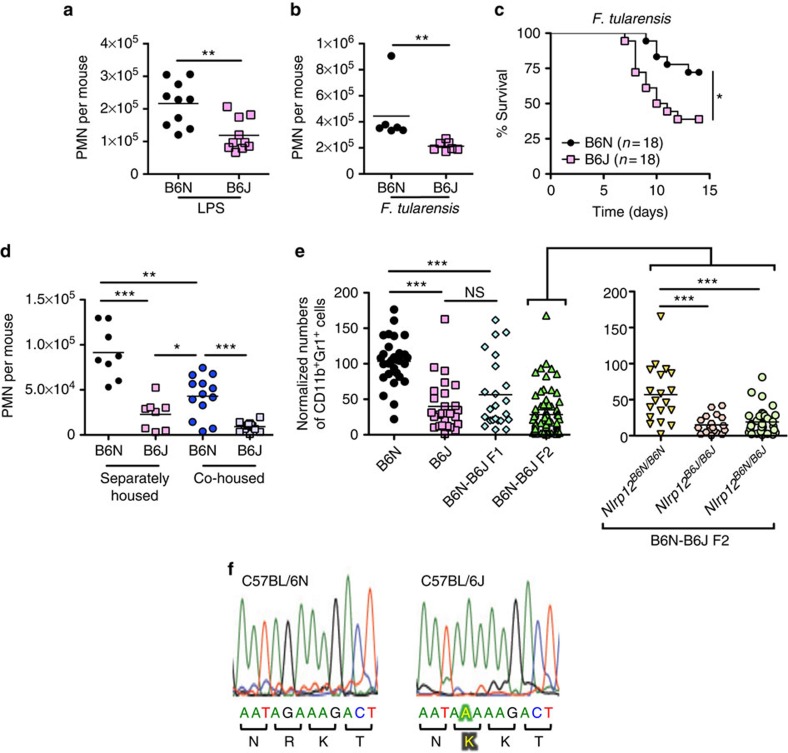
*Nlrp12* mutation in C57BL/6J mice results in defective neutrophil recruitment. (**a**,**b**) C57BL/6N (B6N) and C57BL/6J (B6J) mice were challenged i.n. with LPS (0.5 mg kg^−1^ of body weight) (*n*=10 per group) (**a**), or 5 × 10^3^ colony-forming unit (CFU) of *F. tularensis* LVS (*n*=6 per group) (**b**). Six hours (**a**) or 72 h (**b**) post challenge BAL were performed and the number of Gr1^+^ neutrophils quantified by flow cytometry. (**c**) B6N and B6J mice were challenged i.n. with 5 × 10^3^ CFU of *F. tularensis* LVS and survival monitored. (**d**) B6N and B6J mice were either housed separately or co-housed at a 1:1 ratio for 4 weeks before i.n. challenge with LPS. Neutrophil influx (CD45.2^+^ CD11b^+^ Gr1^+^) into the BAL was determined 6 h post challenge by flow cytometry (*n*=8, separately housed group; *n*≥12, co-housed group). (**e**) B6N, B6J, B6N-B6J F1 and B6N-B6J F2 mice were challenged i.n. with LPS. Six hours post challenge BAL were performed and the number of neutrophils (CD45.2^+^ CD11b^+^ Gr1^+^) quantified by flow cytometry. The *Nlrp12* allele was sequenced for all B6N-B6J F2 mice and cohorts stratified based on their *Nlrp12* genotype (*Nlrp12*^*B6N/B6N*^, *Nlrp12*^*B6N/B6J*^ or *Nlrp12*^*B6J/B6J*^) (*n*=29, B6N; *n*=27, B6J; *n*=23, F1; *n*=73, F2). (**f**) Nucleotide sequence profile of the *Nlrp12* allele in B6N and B6J mice. The highlighted text indicates the presence of a G to A missense polymorphism in C57BL/6J mice leading to the change of an arginine for lysine at position 1034 of NLRP12. *P<0.05, **P<0.01, ***P<0.005, NS, not significant by Mann–Whitney *U*-test (**a**,**b**,**d**,**e**) or log-rank test (**c**).

Although differences in response to acetaminophen-induced hepatotoxicity and endotoxic shock have been noted in C57BL/6 substrains the genetic cause has not been determined^2^,^13^. We evaluated the ability of C57BL/6N and C57BL/6J mice to recruit neutrophils in response to inflammatory stimuli *in vivo*. C57BL/6N and C57BL/6J mice were challenged intranasally (i.n.) with lipopolysaccharide (LPS) or *F. tularensis* live vaccine strain (LVS) and the number of neutrophils in the bronchoalveolar lavage (BAL) quantified by flow cytometry. The total number of neutrophils present in the BAL of C57BL/6J mice was diminished compared with that from C57BL/6N mice for both stimuli (Fig. 1a,b, Supplementary Fig. 1a–f). This C57BL/6 substrain difference was specific to the neutrophilic response, as numbers of CD45.2^+^Gr1^−^ cells were not significantly different between C57BL/6N and C57BL/6J mice challenged i.n. with LPS (Supplementary Fig. 1g,h).

C57BL/6J mice also demonstrated increased mortality in response to i.n. challenge with *F. tularensis* LVS compared with C57BL/6N mice (Fig. 1c). Taken together, these data demonstrate that C57BL/6J mice have diminished inflammatory responses compared with C57BL/6N mice and this translates to increased susceptibility to bacterial infection *in vivo*.

### C57BL/6J *Nlrp12* mutation causes defective neutrophil recruitment

Commensal intestinal bacteria can have profound effects on the host immune system. Ivanov *et al*.^14^ demonstrated that differences in segmented filamentous bacterium (SFB) colonization between C57BL/6 mice from Jackson Laboratories and Taconic Farms influenced Th17 cell differentiation. To assess the possibility that the differences in neutrophilic responses observed between C57BL/6N and C57BL/6J mice was due to differences in intestinal microbiota, C57BL/6N and C57BL/6J mice were co-housed for 4 weeks before i.n. LPS challenge. A significant decrease in neutrophil recruitment in the BAL was still observed in C57BL/6J mice that had been co-housed with C57BL/6N mice (Fig. 1d) suggesting a predominant genetic rather than environmental cause for this phenotypic difference. We did observe that C57BL/6N mice that had been co-housed with C57BL/6J mice had decreased neutrophilic responses compared with separately housed C57BL/6N mice (Fig. 1d) implying that there may be an additional role for microbiota in neutrophil recruitment.

To identify the genetic cause of the neutrophil defect observed in C57BL/6J mice we intercrossed C57BL/6N and C57BL/6J mice (Supplementary Fig. 2a)^15^. We found F_1_ offspring had neutrophil recruitment similar to C57BL/6J mice in response to i.n. LPS challenge, suggesting the C57BL/6J trait was dominant (Fig. 1e). Others have compared the genome between C57BL/6N and C57BL/6J substrains and over 40 SNPs and 30 indels have been identified that differ between the substrains^1^,^2^,^3^,^16^. To determine which genetic loci contribute to the reduced neutrophil recruitment observed in C57BL/6J mice we took a candidate gene approach. F_1_ mice were intercrossed and 73 F_2_ offspring were obtained and analysed by sequencing for the presence of the previously identified SNPs and indels. C57BL/6J mice have been reported to carry a missense polymorphism in chromosome 7 at position 3,222,537 (G to A) leading to the substitution of a lysine for an arginine in the C-terminal leucine-rich repeat domain of NLRP12 (ref. 1), which we confirmed (Fig. 1f and Supplementary Fig. 2b). We genotyped the F_2_ mice for this mutation and found the presence of this missense polymorphism in *Nlrp12* in C57BL/6J mice correlated with defective neutrophil recruitment (Fig. 1e). Analysis of two other genes did not reveal any correlation to diminished neutrophil recruitment in C57BL/6J mice (Supplementary Fig. 2c,d). Consistent with the phenotype of F_1_ mice, F_2_ mice that were heterozygous at the *Nlrp12* locus had neutrophil recruitment similar to C57BL/6J mice in response to i.n. LPS challenge, further suggesting the dominance of the C57BL/6J trait (Fig. 1e).

### Haematopoietic NLRP12 controls neutrophil recruitment

**Figure 2:**
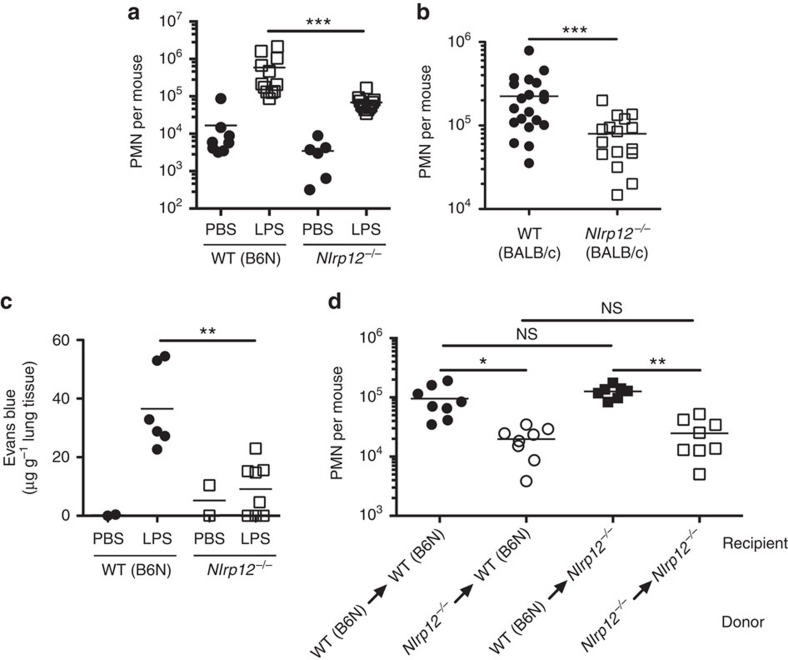
Haematopoietic *Nlrp12* expression is required for effective neutrophil recruitment. (**a**) WT (B6N) or *Nlrp12*^*−/−*^ mice were challenged i.n. with either PBS or LPS (0.5 mg kg^−1^ of body weight). Six hours post challenge BAL were performed and the number of Gr1^+^ cells quantified by flow cytometry (*n*≥6, PBS-treated group; *n*=12, LPS-treated group). (**b**) WT (BALB/cJ) or *Nlrp12*^*−/−*^ mice on a BALB/cJ background were challenged i.n. with LPS (0.0125, mg kg^−1^ of body weight). Six hours post challenge BAL were performed and the number of Gr1^+^ cells quantified by flow cytometry (*n*≥17 per group). (**c**) WT (B6N) or *Nlrp12*^*−/−*^ mice were challenged i.n. with either PBS or LPS (0.5 mg kg^−1^ of body weight). In all, 5.5 h post challenge mice were injected i.v. with Evans blue dye (20 mg kg^−1^). At 6 h post LPS challenge, the amount of extravasated Evans blue dye into the lung was calculated (*n*=2, PBS-treated group; *n*≥6 LPS-treated group). (**d**) Bone marrow chimeras (donor→recipient) were challenged i.n. with LPS (0.5 mg kg^−1^ of body weight). Six hours post challenge, the number of Gr1^+^ cells in the BAL was determined by flow cytometry (*n*=8 per group). *P<0.05, **P<0.01, ***P<0.005, NS, not significant by Mann–Whitney *U*-test (**a**–**c**) or Kruskal–Wallis test with Dunn's multiple comparisons test (**d**).

To specifically determine if NLRP12 was required for effective neutrophil recruitment to inflammatory stimuli, we utilized mice in which *Nlrp12* had been genetically ablated. Similar to C57BL/6J mice, *Nlrp12*^*−/−*^ mice challenged i.n. with LPS displayed significantly diminished neutrophilic influx into the BAL fluid from lungs, as compared with wild-type (WT) C57BL/6N mice (Fig. 2a). *Nlrp12*^*−/−*^ mice backcrossed onto a BALB/cJ background also had a neutrophil recruitment defect in response to intranasal LPS challenge (Fig. 2b). Taken together, these data demonstrate a defect in neutrophil migration in response to LPS in *Nlrp12*^*−/−*^ mice.

In addition to neutrophilic infiltration, vascular leakage has an important role in the pathology of LPS-induced acute lung injury. Therefore, we examined the role of NLRP12 in LPS-induced increase in microvascular permeability. In WT (C57BL/6N) mice, LPS inhalation caused an increase in Evans blue leakage into the lung (Fig. 2c). In contrast, *Nlrp12*^*−/−*^ mice were protected from Evans blue leakage into the lungs compared with WT (C57BL/6N) mice, suggesting diminished vascular leakage in response to inflammatory stimuli (Fig. 2c).

We next sought to determine whether the defect in neutrophil migration in *Nlrp12*^*−/−*^ mice was due to the loss of NLRP12 in the haematopoietic compartment. We generated bone marrow chimeric mice in which NLRP12 deficiency was restricted to either the haematopoietic or non-haematopoietic compartment. WT (C57BL/6N) mice that received *Nlrp12*^*−/−*^ bone marrow had defective neutrophil recruitment to the lungs in response to an i.n. LPS challenge and recapitulated the phenotype observed in NLRP12-deficient mice (Fig. 2d). Conversely, *Nlrp12*^*−/−*^ mice that received WT (C57BL/6N) bone marrow had intact neutrophil recruitment to the lungs in response to an i.n. LPS challenge in comparison to WT (C57BL/6N) mice that received WT (C57BL/6N) bone marrow (Fig. 2d). These results suggest that defective neutrophil recruitment in *Nlrp12*^*−/−*^ mice was due to a deficiency of NLRP12 within the haematopoietic compartment.

### Neutrophil extrinsic NLRP12 regulates neutrophil migration

**Figure 3:**
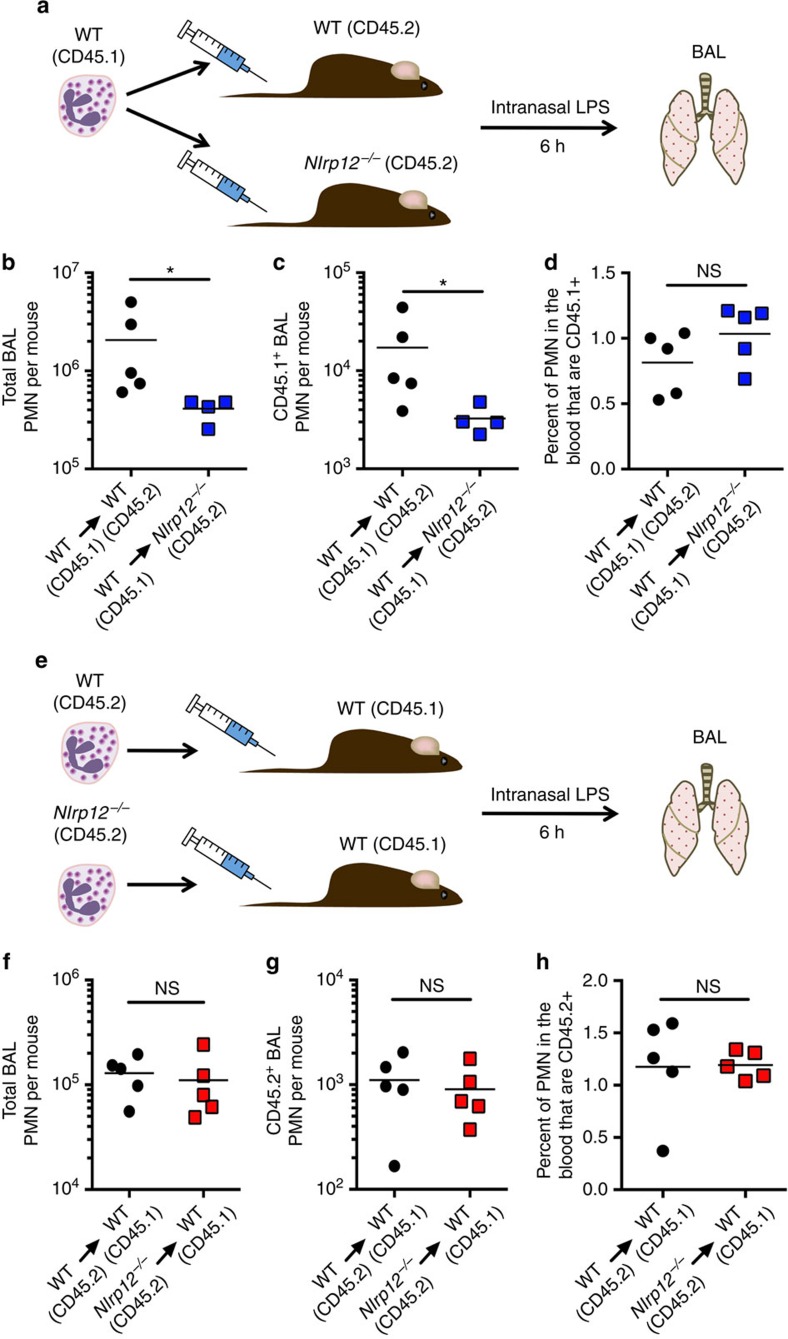
The requirement for NLRP12 in neutrophil recruitment is neutrophil extrinsic. (**a**–**d**) A schematic illustrating the experimental approach is shown (**a**). WT CD45.1^+^ bone marrow was adoptively transferred into CD45.2^+^ WT or CD45.2^+^ *Nlrp12*^*−/−*^ mice. Mice were challenged i.n. with LPS (0.5 mg kg^−1^ of body weight); 6 h post challenge the total number of Gr1^+^ cells (**b**) and the number of CD45.1^+^ Gr1^+^ cells (**c**) in the BAL was determined by flow cytometry. The percentage of CD45.1^+^ Gr1^+^ cells in the blood was also measured by flow cytometry (**d**). (**e**–**h**) A schematic illustrating the experimental approach is shown **e**. CD45.2^+^ WT or CD45.2^+^ *Nlrp12*^*−/−*^ bone marrow was adoptively transferred into CD45.1^+^ WT mice. Mice were challenged i.n. with LPS (0.5 mg kg^−1^ of body weight); 6 h post challenge the total number of Gr1^+^ cells (**f**) and the number of CD45.2^+^ Gr1^+^ cells (**g**) in the BAL was determined by flow cytometry. The percentage of CD45.2^+^ Gr1^+^ cells in the blood was also measured by flow cytometry (**h**). Data are representative of two independent experiments. *P<0.05, NS not significant by Mann–Whitney *U*-test.

To determine if neutrophil intrinsic *Nlrp12* expression was required for appropriate neutrophil recruitment, we utilized an adoptive transfer model. In all, 5 × 10^6^ CD45.1^+^ WT (confirmed to be *Nlrp12*^*B6N/B6N*^) bone marrow cells, as a source of neutrophils, were transferred intravenously into CD45.2^+^ WT (C57BL/6N) or CD45.2^+^ *Nlrp12*^*−/−*^ mice that were then challenged i.n. with LPS. Six hours later the number of CD45.1^+^ neutrophils in the BAL fluid was quantified by flow cytometry (Fig. 3a). As expected, the total number of neutrophils recovered from the BAL of *Nlrp12*^*−/−*^ mice was diminished compared with that from WT (C57BL/6N) recipient mice (Fig. 3b). In addition, the number of CD45.1^+^ WT (*Nlrp12*^*B6N/B6N*^) donor neutrophils in the BAL of *Nlrp12*^*−/−*^ recipient mice was also significantly diminished (Fig. 3c), indicating that normal CD45.1^+^ WT (*Nlrp12*^*B6N/B6N*^) donor neutrophils exhibited defective recruitment in an NLRP12-deficient environment. To confirm that the donor CD45.1^+^ neutrophils had not been specifically eliminated in the *Nlrp12*^*−/−*^ recipient mouse, we compared the frequency of CD45.1^+^ donor neutrophils in the peripheral blood of WT (C57BL/6N) and *Nlrp12*^*−/−*^ recipient mice. A similar frequency of CD45.1^+^ neutrophils were measured in the blood of both WT (C57BL/6N) and *Nlrp12*^*−/−*^ recipient mice (Fig. 3d).

To confirm these findings, we performed the complementary experiment wherein 5 × 10^6^ CD45.2^+^ bone marrow cells from WT (C57BL/6N) or *Nlrp12*^*−/−*^ mice were transferred intravenously into CD45.1^+^ WT (*Nlrp12*^*B6N/B6N*^) mice. Mice were challenged i.n. with LPS and 6 h later the number of donor CD45.2^+^ neutrophils in the BAL fluid was quantified by flow cytometry (Fig. 3e). The total number of neutrophils recovered from the BAL of CD45.1^+^ WT (*Nlrp12*^*B6N/B6N*^) mice that had received bone marrow cells from either WT (C57BL/6N) or *Nlrp12*^*−/−*^ mice was similar (Fig. 3f). Moreover, we saw no difference in the number of donor CD45.2^+^ WT (C57BL/6N) or *Nlrp12*^*−/−*^ neutrophils in the BAL of CD45.1^+^ WT (*Nlrp12*^*B6N/B6N*^) recipient mice or in the frequency of donor CD45.2^+^ WT (C57BL/6N) and *Nlrp12*^*−/−*^ neutrophils in the peripheral blood of CD45.1^+^ WT (*Nlrp12*^*B6N/B6N*^) recipient mice (Fig. 3g,h). These data suggest that *Nlrp12* expression is required in a haematopoietic cell extrinsic to the neutrophil to appropriately direct neutrophil migration to inflammatory stimuli.

### Macrophage NLRP12 is critical for neutrophil recruitment

**Figure 4:**
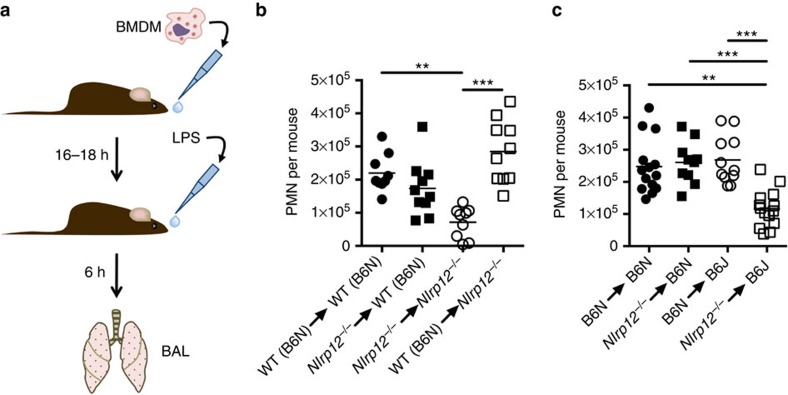
WT (B6N) macrophages rescue the neutrophil defect in C57BL/6J and *Nlrp12*^*−/−*^ mice. (**a**) A schematic illustrating the experimental approach is shown. (**b**) In all, 4 × 10^6^ BMDM from WT (B6N) or *Nlrp12*^*−/−*^ mice were transferred i.n. into either WT (B6N) or *Nlrp12*^*−/−*^ mice (donor→recipient). (**c**) In all, 4 × 10^6^ BMDM from WT (B6N) or *Nlrp12*^*−/−*^ mice were transferred i.n. into either WT (B6N) or WT (B6J) mice (donor→recipient). (**b**,**c**) Mice were rested overnight and then challenged i.n. with LPS (0.5 mg kg^−1^ of body weight). Six hours post challenge the number of Gr1^+^ cells in the BAL was determined by flow cytometry (*n*≥8 per group). **P<0.01, ***P<0.005 by Kruskal–Wallis test with Dunn's multiple comparisons test.

Based on our finding that NLRP12 extrinsic to neutrophils modulated neutrophilic recruitment to inflammatory sites, we hypothesized that macrophage expression of NLRP12 might instruct neutrophil migration indirectly. To examine this hypothesis, we transferred WT (C57BL/6N) or NLRP12-deficient bone marrow-derived macrophages (BMDM) i.n. into WT (C57BL/6N) or *Nlrp12*^*−/−*^ mice. Sixteen hours later mice were challenged i.n. with LPS and then the neutrophilic influx was determined in the BAL fluid (Fig. 4a,b). Transfer of WT (C57BL/6N), but not NLRP12-deficient, BMDM into *Nlrp12*^*−/−*^ mice rescued the neutrophil migration defect *in vivo* in response to LPS challenge (Fig. 4b). These data suggest that NLRP12 expression in macrophages is required to direct appropriate neutrophil migration *in vivo*.

Next, we applied the findings from the NLRP12-deficient mice to C57BL/6J mice that carry a missense polymorphism in *Nlrp12*. To determine if the mechanism for the diminished neutrophil recruitment in C57BL/6J mice is also through defective macrophage function we transferred WT (C57BL/6N) or *Nlrp12*^*−/−*^ BMDM i.n. into C57BL/6N and C57BL/6J recipient mice. Sixteen hours later mice were challenged i.n. with LPS and neutrophil influx determined in the BAL fluid. Transfer of WT (C57BL/6N) BMDM into C57BL/6J recipient mice resulted in the rescue of neutrophil recruitment in response to i.n. LPS as compared with WT (C57BL/6N) recipient mice (Fig. 4c). Importantly, transfer of NLRP12-deficient BMDM into C57BL/6J mice failed to rescue the neutrophil migration defect *in vivo* in response to LPS challenge (Fig. 4c), suggesting that defective NLRP12 function in C57BL/6J macrophages is responsible for the diminished neutrophil recruitment to inflammatory insults *in vivo* in these mice.

### *Nlrp12* ^*−/−*^ mice are more susceptible to bacterial infections

**Figure 5:**
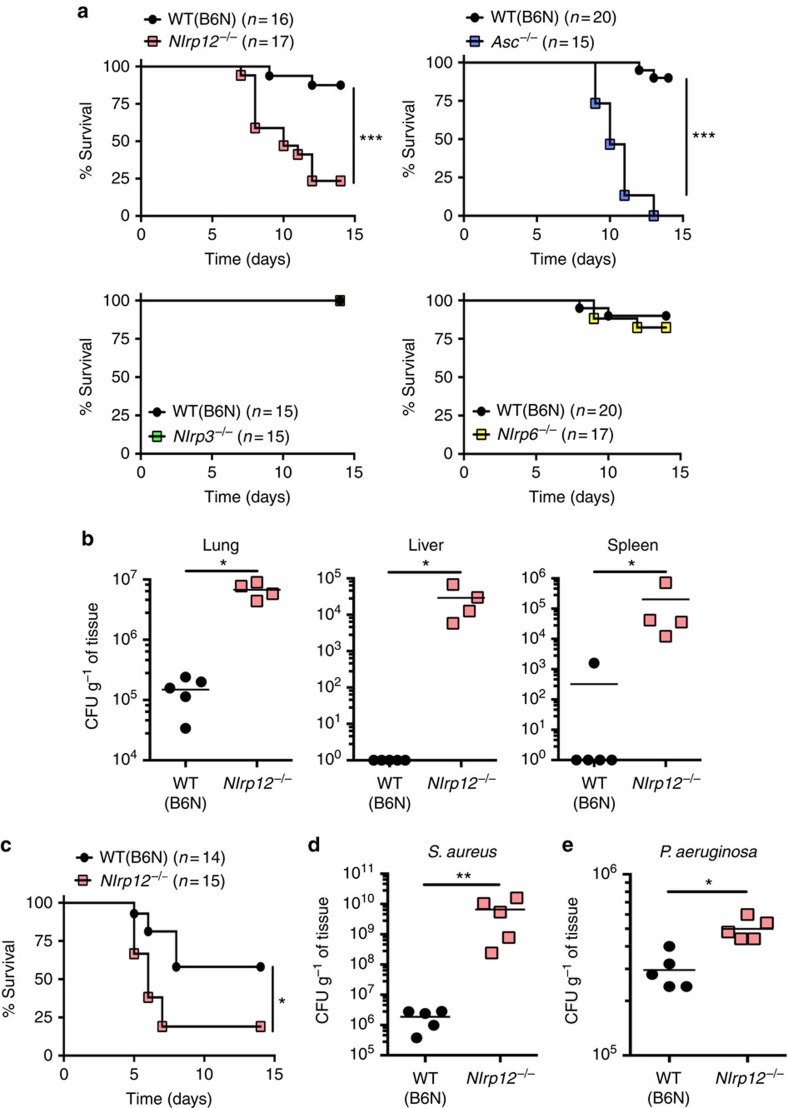
NLRP12 is required for effective control of bacterial pathogens *in vivo*. (**a**) WT (B6N), NLRP12-, NLRP3-, NLRP6- and ASC-deficient mice were infected i.n. with 5 × 10^3^ colony-forming unit (CFU) of *F. tularensis* LVS and survival monitored. (**b**) Nine days post infection with 5 × 10^3^ CFU of *F. tularensis* LVS organs were harvested, homogenized and dilutions plated for enumeration of CFU. (**c**) WT (B6N) and *Nlrp12*^*−/−*^ mice were infected intraperitoneally with 5 × 10^3^ CFU of *F. tularensis* LVS and survival monitored. (**d**,**e**) WT (B6N) and *Nlrp12*^*−/−*^ mice were infected i.n. with either 5 × 10^7^ CFU of *S. aureus* Newman strain (**d**) or 5 × 10^4^ CFU of *P. aeruginosa* PAK strain (E). Sixteen hours (for *S. aureus* infection) or 4 h (for *P. aeruginosa* infection) post infection lungs were harvested, homogenized and dilutions plated for enumeration of CFU. Pooled data from 2–3 independent experiments is shown; *P<0.05, ***P<0.005 by log-rank test (**a**,**c**). Data are representative of 2–3 independent experiments; *P<0.05, **P<0.01 by Mann–Whitney *U*-test (**b**,**d**,**e**).

To determine if the defective neutrophil recruitment observed in *Nlrp12*^*−/−*^ mice affected their ability to control bacterial infections *in vivo*, analogous to the C57BL/6J mice with the missense polymorphism in *Nlrp12*, we utilized a pulmonary *F. tularensis* LVS infection model. ASC-, NLRP3-, NLRP6- and NLRP12-deficient mice were challenged i.n. with a sub-lethal inoculum of *F. tularensis* LVS and survival monitored. As previously reported, mice deficient in ASC (a critical component of several inflammasomes including the AIM2 inflammasome) were highly susceptible to infection with *F. tularensis* LVS with all ASC-deficient mice succumbing to infection by day 13 post infection (Fig. 5a; (refs 17, 18). However, NLRP3- or NLRP6-deficiency did not compromise the ability of mice to survive an i.n. infection with *F. tularensis* LVS (Fig. 5a). In contrast, but consistent with the results for C57BL/6J mice (Fig. 1c), NLRP12-deficient mice were highly susceptible to i.n. infection with *F. tularensis* LVS with >75% of NLRP12-deficient mice succumbing to infection by day 12 post infection (Fig. 5a). The increased susceptibility of *Nlrp12*^*−/−*^ mice to infection with *F. tularensis* LVS compared with WT (C57BL/6N) mice was also reflected by increased bacterial burdens in infected organs 6 and 9 days post infection (Fig. 5b and Supplementary Fig. 3). NLRP12-deficient mice were also more susceptible to an intraperitoneal route of infection with *F. tularensis* LVS compared with WT mice, suggesting that the role of NLRP12 in host defense is not restricted to the lung (Fig. 5c).

In order to determine if NLRP12 had a more global role in host defense against pathogens, we infected NLRP12-deficient mice i.n. with *Staphylococcus aureus* and *Pseudomonas aeruginosa*. Similar to the findings with *F. tularensis* LVS*, Nlrp12*^*−/−*^ mice had significantly higher bacterial burdens of *S. aureus* and *P. aeruginosa* in their lungs compared with WT (C57BL/6N) mice (Fig. 5d,e). Taken together, these data suggest that NLRP12 has an important role in controlling not only intracellular Gram-negative bacteria but also extracellular Gram-negative and Gram-positive pathogens.

### Reduced neutrophil recruitment to *F. tularensis* in *Nlrp12* ^*−/−*^ mice

**Figure 6:**
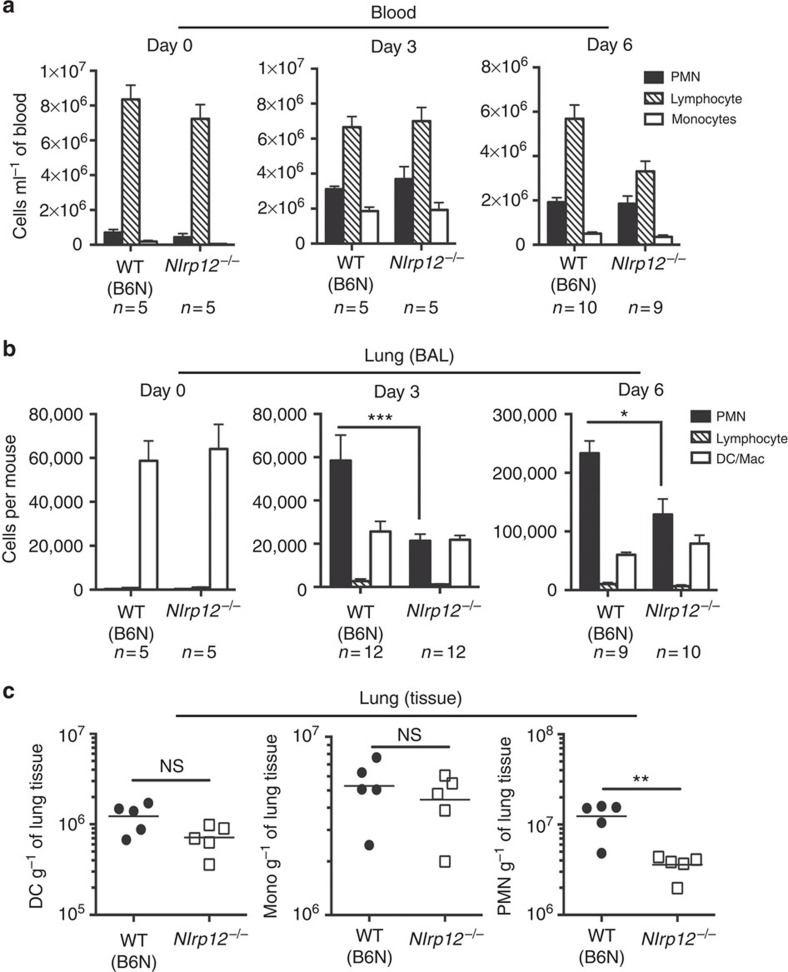
NLRP12 is required for neutrophil recruitment following *F. tularensis* infection. (**a**,**b**) WT (B6N) or *Nlrp12*^*−/−*^ mice were infected i.n. with 5 × 10^3^ colony-forming unit (CFU) of *F. tularensis* LVS. At the indicated time post infection blood was collected and BAL performed. Cell count and differential on blood (**a**) and BAL (**b**) was performed. (**c**) Three days post i.n. infection with 5 × 10^3^ CFU of *F. tularensis* LVS lungs were harvested, prepared into single cell suspensions, stained and analysed by flow cytometry for dendritic cells (CD11b^+^, CD11c^high^, Gr1^-^), macrophages/monocytes (CD11b^+^, CD11c^low^, Gr1^−^) and neutrophils (CD11b^+^, CD11c^−^, Gr1^+^). Pooled data from 2–3 independent experiments is shown (**a**,**b**) or is representative of two independent experiments (**c**). Data are expressed as the mean±s.e.m. (**a**,**b**). *P<0.05, **P<0.01, ***P<0.005, NS, not significant by Mann–Whitney *U*-test.

Neutrophils are rapidly recruited to inflammatory sites through a number of well-defined steps^19^. Neutrophils are initially mobilized from the bone marrow into the peripheral circulation. After receiving the appropriate signals neutrophils interact with the endothelium and undergo tethering, rolling, adhesion, crawling and transendothelial migration. In some instances, neutrophils can additionally exit the tissue parenchyma via transepithelial migration. To define at what point neutrophil migration was disrupted in *Nlrp12*^*−/−*^ mice, we evaluated the *in vivo* mobilization of inflammatory cells in response to infection with *F. tularensis* LVS. Circulating neutrophils were markedly increased at day 3 post infection in both WT (C57BL/6N) and *Nlrp12*^*−/−*^ mice (Fig. 6a). This suggested two things: first that the absence of NLRP12 did not impact the development of neutrophils or second impact their release from the bone marrow into peripheral circulation. In contrast, significantly fewer neutrophils were recovered from the BAL fluid following infection with *F. tularensis* LVS in *Nlrp12*^*−/−*^ mice compared with WT (C57BL/6N) (Fig. 6b and Supplementary Fig. 4).

Movement from the circulation into the alveolar space requires the neutrophils to cross first an endothelial and then an epithelial barrier. To differentiate if neutrophil migration was impaired at the level of transendothelial or transepithelial migration, we assessed the number of neutrophils in the lung parenchyma by flow cytometry. Analogous to the loss of neutrophils in the BAL fluid, we also observed a similar defect in the number of neutrophils recruited across the endothelial barrier into the lung parenchyma of *Nlrp12*^*−/−*^ mice 6 days post infection with *F. tularensis* LVS (Fig. 6c). The impact of NLRP12 deficiency was limited to the neutrophilic response, as the number of parenchymal macrophages and dendritic cells were not significantly different between WT (C57BL/6N) and *Nlrp12*^*−/−*^ mice (Fig. 6c). Taken together, these data suggest that the defect in neutrophil migration observed in *Nlrp12*^*−/−*^ mice infected with *F. tularensis* LVS lies at the level of transendothelial migration out of the vasculature and into the lung parenchyma.

### NLRP12-deficient macrophages have defective CXCL1 production

**Figure 7:**
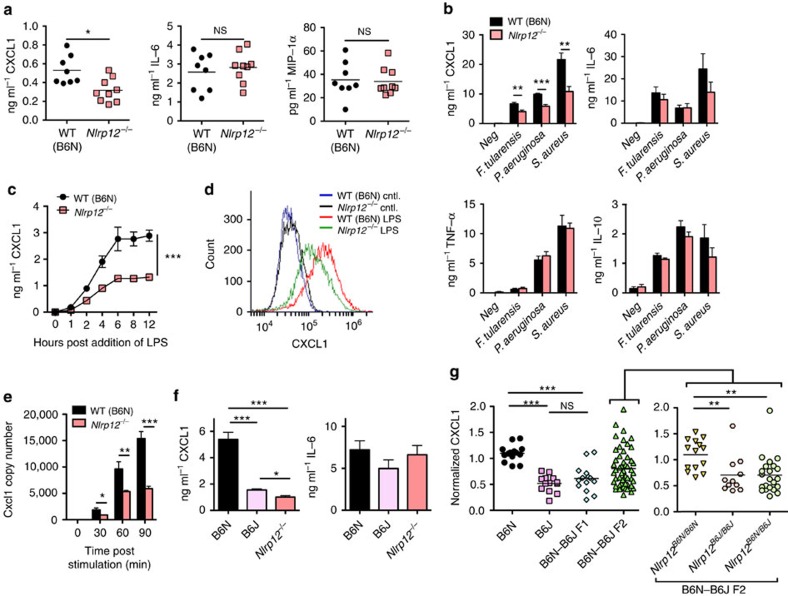
BMDM from C57BL/6J and *Nlrp12*^*−/−*^ mice have defective CXCL1 production. (**a**) WT (B6N) or *Nlrp12*^*−/−*^ mice were infected i.n. with 5 × 10^3^ colony-forming unit of *F. tularensis* LVS. Three days post infection BAL were performed and cytokine levels were determined by ELISA (*n*=8, WT; *n*=9, *Nlrp12*^*−/−)*^. (**b**) BMDM from WT (B6N) and *Nlrp12*^*−/−*^ mice were challenged with either *F. tularensis* LVS, *S. aureus* or *P. aeruginosa*. Eight hours later supernatants were collected and secretion of the indicated cytokine or chemokine quantified by ELISA. (**c**) BMDM from WT (B6N) and *Nlrp12*^*−/−*^ mice were stimulated with LPS (50 ng ml^−1^) for the indicated amount of time; supernatants were collected and assayed for CXCL1 production by ELISA. (**d**) BMDM from WT (B6N) and *Nlrp12*^*−/−*^ mice were left unstimulated or stimulated for 4 h with LPS in the presence of brefeldin A and monensin; intracellular CXCL1 was then assessed by flow cytometry. (**e**) BMDM from WT (B6N) and *Nlrp12*^*−/−*^ mice were stimulated with LPS for the indicated amount of time; *Cxcl1* expression was quantified by real-time PCR. (**f**) BMDM from WT (B6N), WT (B6J) and *Nlrp12*^*−/−*^ mice were stimulated with LPS for 8 h; supernatants were collected and assayed for the indicated cytokines by ELISA. (**g**) BMDM from B6N, B6J, B6N-B6J F1 and B6N-B6J F2 mice were challenged with LPS for 8 h; supernatants were collected and CXCL1 secretion assessed by ELISA. The *Nlrp12* allele was sequenced for all B6N-B6J F2 mice and cohorts stratified based on their *Nlrp12* genotype (*Nlrp12*^*B6N/B6N*^, *Nlrp12*^*B6N/B6J*^ or *Nlrp12*^*B6J/B6J*^) (*n*=14, B6N; *n*=13, B6J; *n*=14, F1; *n*=52, F2). Pooled data from three (**b**,**c**) independent experiments are depicted or are representative of two (**d**) or three (**e**,**f**) independent experiments. (**b**,**c**,**e**,**f**) Data are expressed as the mean±s.e.m. *P<0.05, **P<0.01, ***P<0.005, NS, not significant by Mann–Whitney *U*-test (**a**,**g**), Student's *t*-test (**b**,**e**,**f**) or two-way analysis of variance analysis (**c**).

To further characterize the role of NLRP12 in the response to *F. tularensis* LVS infection, we next asked what the mechanism is by which NLRP12 acts in macrophages to direct neutrophil migration. We examined the induction of cytokines and chemokines in BAL fluid from WT (C57BL/6N) and NLRP12-deficient mice 3 days post infection. Whereas IL-6, tumour-necrosis factor (TNFα), IL-17A, macrophage inflammatory protein (MIP)-1α, IL-18 and IL-1β levels were not different in *Nlrp12*^*−/−*^ mice compared with WT (C57BL/6N) mice, the neutrophil chemotactic chemokine CXCL1 levels were significantly diminished in the BAL fluid from *Nlrp12*^*−/−*^ mice compared with WT (B6N) (Fig. 7a and Supplementary Fig. 5a).

Consistent with our *in vivo* findings of diminished CXCL1 in BAL from *Nlpr12*^*−/−*^ mice following infection with *F. tularensis* LVS, we observed that *Nlpr12*^*−/−*^ BMDM secreted significantly less CXCL1 in response to *F. tularensis* LVS, *S. aureus* and *P. aeruginosa* (Fig. 7b). We observed a similar defect in CXCL1 production in the absence of NLRP12 in bone marrow-derived dendritic cells (BMDC) challenged with *F. tularensis* LVS, *S. aureus* or *P. aeruginosa* (Supplementary Fig. 5b). However, the production of IL-6, TNFα and IL-10 in response to infection with *F. tularensis* LVS, *S. aureus* or *P. aeruginosa* was similar between WT (C57BL/6N) and *Nlrp12*^*−/−*^ BMDM (Fig. 7b).

To determine if the defect in CXCL1 production was restricted to infection with viable bacteria, we challenged BMDM from WT (C57BL/6N) and *Nlrp12*^*−/−*^ mice with a variety of toll-like receptor (TLR) agonists. Whereas NLRP12-deficient BMDM produced amounts of IL-6 and TNFα similar to those of WT (C57BL/6N) BMDM, NLRP12-deficient cells released less CXCL1 in response to all TLR agonists tested (Fig. 7c and Supplementary Fig. 5c). In support of the enzyme-linked immunosorbent assay (ELISA) data, intracellular cytokine staining also demonstrated less CXCL1 production in NLRP12-deficient BMDM compared with that in WT (C57BL/6N) challenged with LPS (Fig. 7d). Examination of *Cxcl1* expression by quantitative reverse transcription–PCR also found diminished *Cxcl1* expression in NLRP12-deficient BMDM compared with WT (C57BL/6N) BMDM stimulated with LPS (Fig. 7e). Evaluation of mitogen-activated protein kinases (MAPK), canonical NF-κB and non-canonical NF-κB pathways in response to LPS stimulation did not reveal any substantial differences between BMDM from WT (C57BL/6N) and *Nlrp12*^*−/−*^ mice (Supplementary Fig. 5d–g). Taken together, these data suggest that NLRP12 was specifically required for CXCL1 production and that this regulation occurred at the level of *Cxcl1* transcript abundance.

Vladimer *et al*.^10^ have shown that *Y. pestis* can activate macrophage caspase-1 in an NLRP12-dependent manner and result in the generation of IL-1β and IL-18. To determine if NLRP12 was required to trigger inflammasome activation in response to *F. tularensis* LVS, *S. aureus* or *P. aeruginosa*, we measured IL-1β secretion and caspase-1 activation from BMDM from WT (C57BL/6N) and *Nlrp12*^*−/−*^ mice challenged with *F. tularensis* LVS, *S. aureus* or *P. aeruginosa*. The production of IL-1β and caspase-1 activation between WT (C57BL/6N) and NLRP12-deficient BMDM following challenge with *F. tularensis* LVS, *S. aureus* or *P. aeruginosa* were similar (Supplementary Fig. 6a,b). Similarly, no defect in the production of IL-1β in the absence of NLRP12 was observed in BMDC challenged with *F. tularensis* LVS, *S. aureus* or *P. aeruginosa* (Supplementary Fig. 6c). These data demonstrate that NLRP12 did not participate in the assembly or activation of an inflammasome in response to *F. tularensis* LVS, *S. aureus* or *P. aeruginosa*, and that the increased susceptibility of *Nlrp12*^*−/−*^ mice to infection with these organisms was independent of caspase-1 activation.

### Diminished CXCL1 production in C57BL/6J macrophages

We next examined if BMDM from C57BL/6J also exhibited defects in CXCL1 production in response to LPS. Similar to *Nlrp12*^*−/−*^ BMDM, CXCL1 production from C57BL/6J BMDM stimulated with LPS was significantly diminished compared with C57BL/6N BMDM. Furthermore, CXCL1 production from C57BL/6J BMDM was diminished to a similar level as BMDM from *Nlrp12*^*−/−*^ mice (Fig. 7f). Again consistent with the findings for *Nlrp12*^*−/−*^ BMDM, in response to LPS stimulation C57BL/6J and C57BL/6N BMDM produced equivalent levels of IL-6, TNFα, IL-1α and IL-10 (Fig. 7f and Supplementary Fig. 7a). Examination of *Cxcl1* expression by quantitative reverse transcription–PCR also found diminished *Cxcl1* expression in C57BL/6J BMDM compared with C57BL/6N BMDM stimulated with LPS (Supplementary Fig. 7b). Furthermore, LPS stimulation of TLR4-expressing HEK293 cells (HEK-TLR4) transfected with *Nlrp12*^*B6N*^ resulted in significantly greater CXCL1 production compared with stimulation of those transfected with *Nlrp12*^*B6J*^ (Supplementary Fig. 7c,d).

To determine if the missense mutation in C57BL/6J *Nlrp12* contributed to the diminished CXCL1 production observed in C57BL/6J BMDM we analysed CXCL1 production from BMDM in response to LPS from 52 F_2_ offspring that were then segregated by *Nlrp12* genotype (Fig. 7g). The presence of the C57BL/6J missense polymorphism in *Nlrp12* correlated with diminished CXCL1 production by the F_2_ BMDM (Fig. 7g). BMDM from F_1_ mice and F_2_ mice that were heterozygous at the *Nlrp12* locus had CXCL1 production similar to C57BL/6J mice in response to LPS challenge, further suggesting the dominance of the C57BL/6J trait (Fig. 7g).

## Discussion

Our work demonstrates differences in inflammatory responses between C57BL/6N and C57BL/6J mouse substrains with C57BL/6J mice having a failure of neutrophil recruitment to a wide variety of inflammatory stimuli compared with the related C57BL/6N substrain. C57BL/6J mice carry a missense polymorphism in chromosome 7 at position 3,222,537 (G to A) leading to the change of an arginine for lysine in the C-terminal leucine-rich repeat domain of NLRP12 (ref. 1). Our findings show that this *Nlrp12* SNP is associated with the difference in neutrophil recruitment found between C57BL/6N and C57BL/6J substrains and that NLRP12 is required in macrophages to direct neutrophil recruitment. This failure by macrophages, both the NLRP12-deficient and the NLRP12 missense mutant, to recruit neutrophils is associated with defective production of the neutrophil chemoattractant chemokine CXCL1. These data suggest a single seemingly conservative amino acid change within NLRP12 can render it dysfunctional and profoundly impact a wide range of immune responses both *in vitro* and *in vivo*. Our data suggest that C57BL/6J mice carrying the *Nlrp12* missense polymorphism may be inappropriate controls to study NLRP12 function. Furthermore, the use of C57BL/6J mice, as well as knockout mice backcrossed onto the C57BL/6J substrain, in studies that depend upon intact neutrophilic responses or that assess control of infectious pathogens may significantly confound their interpretation due to the presence of a dysfunctional NLRP12.

The literature surrounding NLRP12 is complex as several lines of investigation suggest contradictory roles for NLRP12 in response to infection with bacterial pathogens. NLRP12 has been shown to be important for host defense against *Y. pestis* through the production of caspase-1-dependent IL-18 (ref. 10). In contrast, NLRP12 has a detrimental role in infection with *S. typhimurium* through negative regulation of NF-κB and MAPK^11^. In the current study, we demonstrate a novel role for NLRP12 in host defense against infection with *F. tularensis* LVS, *S. aureus* and *P. aeruginosa*. In contrast to the findings with *Y. pestis*^10^, we did not observe a defect in caspase-1 mediated IL-1β production in *Nlrp12*^*−/−*^ mice in response to *F. tularensis* LVS, *S. aureus* and *P. aeruginosa*, suggesting that NLRP12 did not regulate inflammasome activation in response to these pathogens. However, *Nlrp12*^*−/−*^ mice failed to control replication of *F. tularensis* LVS, *S. aureus* and *P. aeruginosa in vivo*, a failure that correlated with a defective recruitment of neutrophils to the site of infection. Mobilization of neutrophils from the bone marrow into the peripheral circulation was intact in the absence of NLRP12 in mice infected i.n. with *F. tularensis* LVS. However, these newly recruited neutrophils were defective in their ability to exit from the peripheral circulation and migrate into the lung parenchyma in *Nlrp12*^*−/−*^ mice, thereby suggesting a defect in transendothelial migration. Consistent with our findings a study by Cai *et al*.^20^demonstrated that NLRP12-deficient mice were more susceptible to pulmonary infection with *K. pneumoniae in vivo* and that this was in part due to defective CXCL1 production. Interestingly, a study by Arthur *et al*.^21^ demonstrated an *in vitro* migration defect of NLPR12-deficient neutrophils to CXCL1. In contrast, Zamoshnikova *et al*.^22^ demonstrated an increase in neutrophil migration *in vitro* to the higher of two doses of CXCL1, but not in migration to *Leishmania major*. Although our *in vivo* assays did not reveal a neutrophil intrinsic role for NLRP12, the findings of Arthur *et al*. and Zamoshnikova *et al*. suggest there may be an additional neutrophil intrinsic role for NLRP12 (refs 21, 22).

Perivascular macrophages in a variety of tissues are in close proximity to blood vessels and potently express CXCL1 and CXCL2 in response to challenge with infectious stimuli^23^. Chemokines secreted by perivascular macrophages are thought to create the precise cytokine milieu triggering both the adhesion and transmigration of neutrophils out of the blood stream^23^. Consistent with a role for a perivascular macrophage-like cell, local pulmonary reconstitution of *Nlrp12*^*−/−*^ mice with WT (C57BL/6N) macrophages, but not *Nlrp12*^*−/−*^ macrophages, corrected defective neutrophil recruitment to the lungs in response to LPS. These findings suggest that the NLRP12-dependent production of a chemotactic signal from local macrophages is a necessity for protective neutrophil recruitment in response to inflammatory stimuli *in vivo*. In further support of this role for macrophage-derived NLRP12, we observed a specific defect in the ability of NLRP12-deficient macrophages to secrete CXCL1 in response to a variety of pathogens and TLR agonists. These findings are consistent with a previous study in which diminished CXCL1 production was observed in NLRP12-deficient dendritic cells stimulated with the TLR2 agonist Pam3Cys4 compared with WT dendritic cells^8^.

In conclusion, our results demonstrate that NLRP12 is critical in the regulation of neutrophil recruitment to inflammatory triggers and that this is through control of the production of CXCL1 by local tissue macrophages. This neutrophil-extrinsic mechanism in turn is important for host defense against a variety of bacterial pathogens. Furthermore, a missense mutation in *Nlrp12* in C57BL/6J mice renders them with defective NLRP12 function and hence diminished neutrophil recruitment to inflammatory stimuli *in vivo*. It is important to note that although the mutation in *Nlrp12* in C57BL/6J mice results in defective neutrophil migration *in vivo*, it remains to be seen if other roles for NLRP12 are affected by this missense mutation. Further studies are also needed to define how the C57BL/6J missense mutation in NLPR12 affects its structure and binding to potential ligands and downstream signalling molecules. In addition, the relevance of these findings to human NLRP12 function will require further investigation.

## Methods

### Mice

The generation of NLRP12-, NLRP6-, NLRP3- and ASC-deficient mice has been described previously^21^,^24^,^25^. Mice were backcrossed onto the C57BL/6N genetic background for at least nine generations. *Nlrp12*^*−/−*^ mice were backcrossed onto a BALB/cJ background for eight generations. C57BL/6N mice were purchased from National Cancer Institute and used as WT controls unless otherwise stated; BALB/cJ and C57BL/6NJ mice were purchased from Jackson Laboratories. CD45.1^+^ C57BL/6 mice (B6-Ly5.2/Cr) were purchased from National Cancer Institute. Both male and female mice between 6 and 12 weeks of age were used, however individual experiments utilized age and sex matched animals. The Institutional Animal Care and Use Committee at the University of Iowa approved all protocols used in this study. Bone marrow chimeras were generated as described^26^. Reconstitution was >92% in *Nlrp12*^*−/−*^ mice reconstituted with WT bone marrow and 89% in WT mice reconstituted with *Nlrp12*^*−/−*^ bone marrow.

### Bacterial strains and growth conditions

*F. tularensis* ssp. *holarctica* LVS was obtained from American Type Culture Collection (ATCC 29684). *F. tularensis* LVS was grown on Difco cysteine heart agar supplemented with 9% sheep red blood cells (SRBC) for 48 h at 37 °C. For *in vivo* studies, bacteria were grown overnight in modified Mueller-Hinton (MMH) broth (Becton Dickinson) supplemented with 1% (wt/vol) glucose, 0.025% ferric pyrophosphate, and 2% IsoVitaleX and then subcultured for 4 h before use. *F. tularensis* LVS expressing green fluorescent protein (GFP)^27^ were grown on Difco cysteine heart agar supplemented with 9% SRBC and 25 μg ml^−1^ spectinomycin for 48 h at 37 °C; bacteria were then cultured in supplemented MMH broth with 25 μg ml^−1^ spectinomycin at 37 °C overnight, and subcultured for 4 h in supplemented MMH broth. *P. aeruginosa* PAK strain was obtained from Barbara Kazmierczak (Yale University). *P. aeruginosa* was cultured in lysogeny broth broth overnight at 37 °C and then subcultured for 4 h before use. *S. aureus* Newman strain was obtained from William Nauseef (University of Iowa); *S. aureus* was cultured overnight in tryptic soy broth at 37 °C and then subcultured for 4 h before use.

### *In vivo* infection and LPS challenge

For *in vivo* infections mice were anaesthetized with ketamine/xylazine and infected i.n. with the indicated number of bacteria resuspended in 50 μl of PBS. Alternatively, mice were injected intraperitoneally with 5 × 10^3^ colony-forming unit of *F. tularensis* LVS resuspended in 200 μl of PBS. Mice were monitored every 12–24 h for lethality; mice found to be in a moribund state for more than 4 h were considered terminal and euthanized. Bacterial burdens in the lung, spleen and liver of infected mice were determined at the indicated times post infection by dilution plating of tissue homogenates onto Difco cysteine heart agar supplemented with 9% SRBC for *F. tularensis* LVS, lysogeny broth for *P. aeruginosa* or TSA for *S. aureus*. For *in vivo* LPS challenge mice were anaesthetized with ketamine/xylazine and challenged i.n. with the indicated amount of LPS (*E. coli* serotype 0111:B4; Invivogen) in 50 μl of PBS. BAL was performed on a subset of mice at the indicated time by cannulation of the trachea and lavage with either 3 ml (for cell assessment) or 1 ml (for cytokine determination) of PBS^28^. Red blood cells were lysed and total nucleated cell counts were obtained using a haemocytometer. Cytospin slides were prepared by Wright-Giemsa staining with HEMA 3 (Fisher) and numbers of neutrophils, lymphocytes and dendritic cells/macrophages quantified. Alternatively, BAL cell differentials were determined by flow cytometry. At the indicated time, post infection blood was collected and blood count /differential was performed by the Clinical Pathology Laboratory at Iowa State University.

### Microvascular permeability

We assessed microvascular permeability through extravasation of Evans blue dye. Thirty minutes before euthanasia, mice were injected intravenously with Evans blue dye (20 mg kg^−1^; Sigma). Lungs were perfused with PBS to remove intravascular Evans blue dye. Lungs were harvested and the Evans blue dye extracted as previously described^29^. Briefly, lungs were harvested, homogenized and incubated overnight with 16.7% formamide in PBS at 37 °C. Lung homogenates were filtered through a 70 μm mesh and plated into 96-well flat bottom plates. Absorbance at 620 and 740 nm were measured and used to correct for the presence of haeme pigments using the following equation: *A*_620_ (corrected)=*A*_620_−(1.426 × *A*_740_+0.03) (refs 29, 30). The amount of extravasated Evans blue dye was then calculated against a standard curve.

### Adoptive cell transfer

For adoptive transfer mice were injected intravenously in the tail vein with 5 × 10^6^ bone marrow cells from WT or *Nlrp12*^*−/−*^ mice in PBS into congenically mismatched recipients before intranasal LPS challenge. Adoptive transfer of BMDM was accomplished by intranasal delivery of 4 × 10^6^ BMDM derived from either donor WT or *Nlrp12*^*−/−*^ mice into the corresponding recipient; 16–18 h later mice were i.n. challenged with LPS.

### Flow cytometry

Single cell suspensions of lungs prepared by pressing tissues through wire mesh screens or BAL were used and live cells enumerated using trypan blue exclusion. In all, 0.1–1 × 10^6^ cells per well were stained in a 96-well plate and blocked with 2% rat serum or Fc Block (anti-mouse CD16/CD32; clone 2.4G2; 1:500; BioLegend) in FACS Buffer for 30 min at 4 °C. Following blocking, BAL cells and lung homogenate cells were stained for 30 min at 4 °C with anti-CD45.2 (PerCP-Cy5.5 or PE-Cy7; clone 104; 1:100; BioLegend), anti-CD45.1 (FITC or PE; clone A20; 1:100; Biolegend), anti-CD11b (FITC, PE or PE-Cy7; clone M1/70; 1:400; eBioscience), anti-CD11c (PerCP-Cy5.5; clone N418; 1:200; eBioscience), anti-Gr1 (APC or FITC; clone RB6-8C5 or 1A8; 1:200; eBioscience) and anti-F4/80 (PE; clone BM8; 1:100; eBioscience) as described. Cells were subsequently fixed with FACS lysis buffer (BD Biosciences) per manufacturer's instructions and resuspended in PBS. For CXCL1 intracellular staining BMDMs were left unstimulated or stimulated for 4 h with LPS (50 ng ml^−1^) in the presence of brefeldin A (5 μg ml^−1^; BioLegend) and monensin (2 μM; eBioscience). Cells were permeabilized and stained for the presence of intracellular CXCL1 using a biotinylated anti-mouse CXCL1 antibody (clone BAF453; 1:100; R&D systems). Secondary staining was accomplished with streptavidin PE (eBioscience). Data were acquired on a BD LSR II and analysed using FlowJo Software (Treestar, Inc.).

### *In vitro* stimulation of macrophages and dendritic cells

BMDM and BMDC^31^,^32^ were either left unprimed or primed with 50 ng ml^−1^ LPS for 4 h and then infected with *F. tularensis* LVS (MOI 50:1), *P. aeruginosa* PAK strain (MOI 1:1), or *S. aureus* Newman strain (MOI 1:1) for 8 h and supernatants collected and assayed for cytokine levels by ELISA. For immunoblotting, cells were lysed in lysis buffer (50 mM Tris·HCl, 5 mM EDTA, 150 mM NaCl, 1% Triton X-100 and a protease inhibitor mixture (Roche)) and stored at −80 °C until analysed. Proteins were separated on a NuPAGE gel (Invitrogen) and transferred to a polyvinylidene difluoride membrane by electroblotting^33^. To detect caspase-1, rabbit polyclonal anti-mouse caspase-1 p10 antibody (sc-514; 1:100; Santa Cruz Biotechnology) was used. TLR agonists were obtained from InvivoGen and used to stimulate BMDM as follows: LPS, 50 ng ml^−1^; lipoteichoic acid (LTA), 5 μg ml^−1^; FSL1 (Pam2CGDPKHPKSF), 1 μg ml^−1^; heat-killed *Listeria monocytogenes* (HKLM), 1 × 10^8^ cells ml^−1^; Pam3csk, 1 μg ml^−1^; and R848, 4 μg ml^−1^. Cells were stimulated for 8 h or the indicated time and supernatants collected and assayed for cytokine levels by ELISA. Cell lysates were used for immunoblotting; the following antibodies from Cell Signaling Technology were utilized at 1:1,000: phospho-p38 MAPK (Thr180/Tyr182, product #4511, clone D3F9); total p38 MAPK (product #8690, clone D13E1); phospho-p44/42 MAPK (Thr202/Tyr204, product #4370, clone D13.14.4E); total p44/42 MAPK (product #4695, clone 137F5), phospho-SAPK/JNK (Thr183/Tyr185, product #4668, clone 81E11); total SAPK/JNK (product #9252, polyclonal); phospho-IκBα (Ser32, product #2859, clone 14D4); total IκBα (product #4814, clone L35A5); phospho-NF-κB p65 (Ser536, product #3033, clone 93H1); total NF-κB p65 (product #8242, clone D14E12); NF-κB2 p100/p52 (product #4882, clone 18D10); and α-tubulin (product #2125, clone 11H10).

### Cytokine ELISA

Culture supernatants and BAL samples were assayed for IL-1β, MIP-1α, TNFα, IL-6, IL-10, IL-18, IL-17A and CXCL1 by ELISA. Antibody pairs for the IL-1β (MAB401 and BAF401; clone 30311 and polyclonal; R&D Systems; 8 and 4 μg ml^−1^, respectively) and CXCL1 (mouse: MAB453 and BAF453; clone 48415 and polyclonal; R&D Systems; 8 and 0.4 μg ml^−1^, respectively; human: MAB275, clone 20326, 4 μg ml^−1^; BAF275, polyclonal, 40 ng ml^−1^) and CCL3/ MIP-1α (Quantikine ELISA kit, Product #MMA00) ELISAs were from R&D Systems. Antibody pairs for IL-1α (capture: product #14-7011-85, clone ALF-161; detection: product #13-7111-85, polyclonal), TNFα (capture: product #14-7325-85, clone 1F3F3D4; detection: product #13-7326-85, clones MP6-XT22 and MP6-XT3), IL-6 (capture: product #14-7061-85, clone MP5-20F3; detection: product #13-7062-85, clone MP5-32C11), IL-10 (capture: product #14-7101-85, clone JES5-16E3; detection: product #13-7102-85, clone JES5-2A5) and IL-17A (capture: product #14-7175-85, clone eBio 17CK15A5; detection: product #13-7177-85, clone eBio17B7) were from eBiosciences and used at a 1:250 dilution. Antibody pairs for IL-18 were from MBL International (capture: product #D047-3, clone 74, 1:1,000; detection: product #D048-6, clone 93-10C, 1:2,000).

### Transfection

To prepare vectors with B6N and B6J *Nlrp12* variants we mutated *Nlrp12*^*B6J*^ in the pCMV6-AC-GFP vector (Origene Technologies) to *Nlrp12*^*B6N*^ using the Quickchange II XL Site-Directed Mutagenesis Kit (Agilent Technologies) according to the manufacturer's instructions and the duplex primers 5′-GCTGTTTGGGATGGACCTGAATAGAAAGACTCACAGGAGGATGGC-3′ with the reverse complement (Integrated DNA Technologies). The sequence of all *Nlrp12*^*B6N*^- and *Nlrp12*^*B6J*^-containing vectors were validated by Sanger sequencing (Iowa Institute of Human Genetics, Genomics Division). TLR4-expressing HEK293 cells (HEK-TLR4) (ref. 34) were a generous gift from Dr Jerrold Weiss at the University of Iowa. HEK-TLR4 cells were transfected with 2 μg of the GFP-tagged *Nlrp12*^*B6N*^ or *Nlrp12*^*B6J*^ constructs using Lipofectamine 3000 (Thermo Fisher Scientific) according to the manufacturer's instructions. Twenty-four hours after transfection, cells were stimulated for 16 h with 100 ng ml^−1^ LPS. Supernatants were used for ELISA and cell lysates were used for immunoblotting. Anti-turboGFP (OTI2H8) from Origene Technologies was used to detect the GFP tag in transfected cells.

### Real-time PCR and sequencing

Complementary DNA was generated at the indicated time post stimulation with LPS and real-time PCR performed using PerfeCTa SYBR Green Fast Mix (Quanta) and RT2 quantitative PCR primers (Qiagen) for *Cxcl1*. For quantification, standards were generated by cloning the PCR product into the pCR 2.1 vector using TOPO TA cloning kit (Life Technologies).

To identify genetic differences between C57BL/6N and C57BL/6J mice the SNP for *Nlrp12* and indels for *Crb1* and *Nnt* genes were PCR amplified using the following primers. *Nlrp12*: forward primer (5′-CTGGGAGCTGGATTT-3′), reverse primer (5′-TATCCTGGTCGGCTTCATTC-3′); *Crb1*: forward primer (5′- CGAGTGGACTTTGTGCTCACC-3′), reverse primer (5′- TTGACAATCCGAAAGGCCTGC-3′); *Nnt* common primer (5′-GTAGGGCCAACTGTTTCTGCATGA-3′), WT primer (5′-GGGCATAGGAAGCAAATACCAAGTTG-3′) and mutant primer (5′-GTGGAATTCCGCTGAGAGAACTCTT-3′)^35^. Once amplified SNP and indel differences were either identified by Sanger sequencing (*Nlrp12* and *Crb1*) or by running PCR products on a 1% agarose gel (*Nnt*).

### Data availability

The data that support the findings of this study are available from the corresponding author upon request.

## Additional information

**How to cite this article:** Ulland, T. K. *et al*. *Nlrp12* mutation causes C57BL/6J strain-specific defect in neutrophil recruitment. *Nat. Commun.* **7,** 13180 doi: 10.1038/ncomms13180 (2016).

## Supplementary Material

Supplementary Information Supplementary Figures 1-7
