# Supplementary material for: Nlrp12 mutation causes C57BL/6J strain-specific defect in neutrophil recruitment
Source: Nat Commun. 2016 Oct 25;7:13180. doi: 10.1038/ncomms13180 (PMC5093323; doi:10.1038/ncomms13180)
Supplement: Supplementary Information — Supplementary Figures 1-7 [file ncomms13180-s1.pdf]

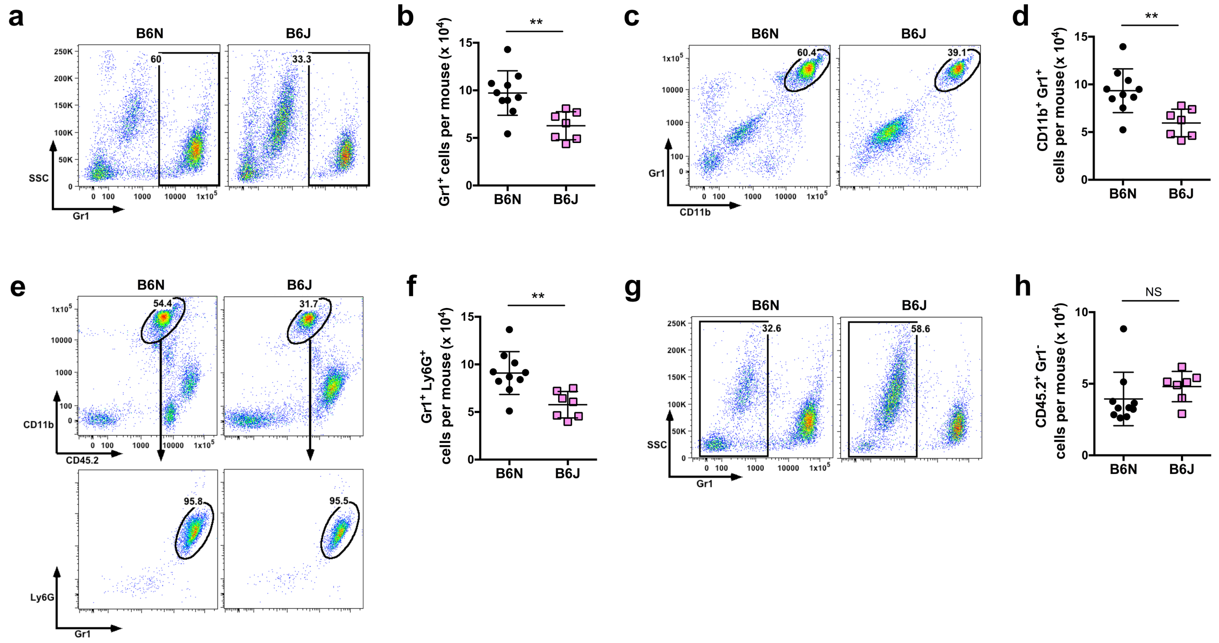

Supplementary Fig. 1. C57BL/6J mice have defective neutrophil recruitment in response to LPS. C57BL/6N and C57BL/6J mice were i.n. challenged with LPS (0.5 mg kg^{-1} of body weight). 6 h post challenge BAL were harvested and cells analyzed by flow cytometry. (**a**) Representative gating and (**b**) absolute numbers for cells stained by anti-Gr1 antibody. (**c**) Representative gating and (**d**) absolute numbers for CD11b$^+$Gr1$^+$ cells gated on the CD45.2$^+$ population. (**e, f**) BAL were stained and gated on the CD45.2$^+$CD11b$^+$ population followed by gating on Gr1$^+$Ly6G$^+$ cells; (**e**) representative gating and (**f**) absolute numbers are shown. (**g**) Representative gating and (**h**) absolute numbers for CD45.2$^+$Gr1$^-$ cells. ** $p < 0.01$, N.S. not significant by Mann-Whitney U test.

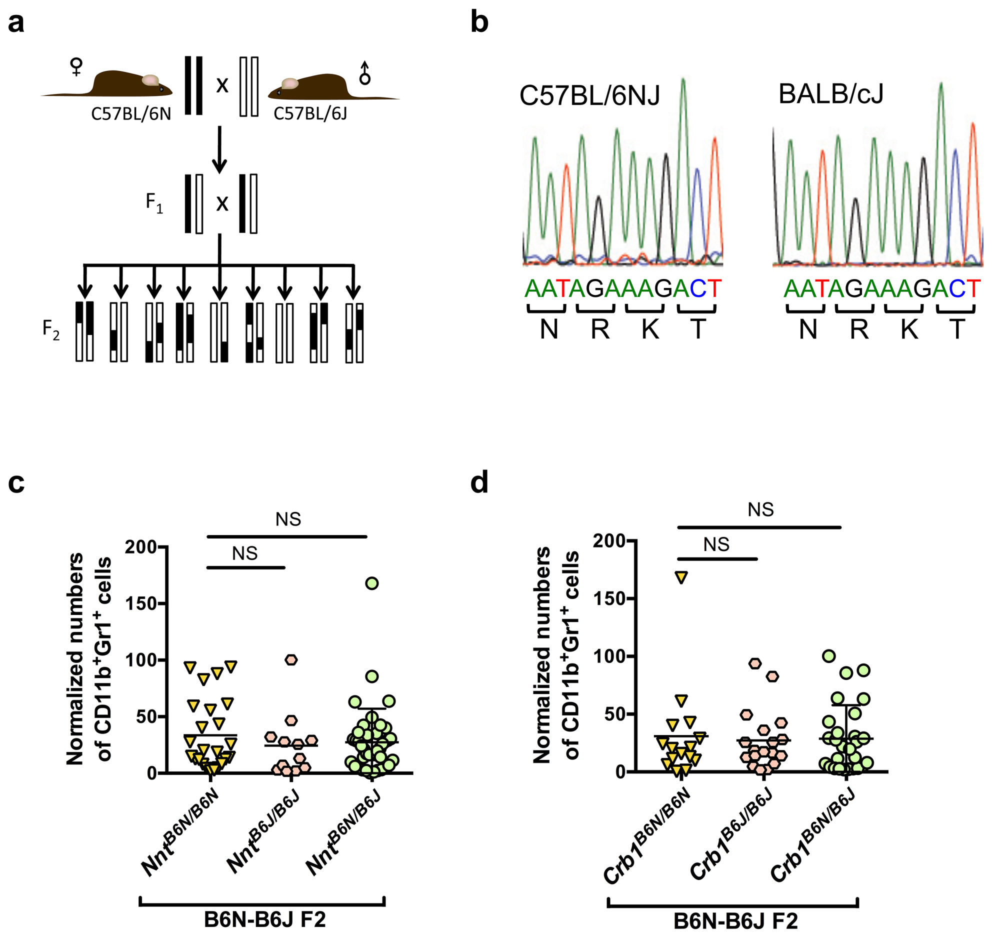

Supplementary Fig. 2. ***Nlrp12*** **missense mutation in C57BL/6J mice results in defective neutrophil recruitment in response to LPS.** (**a**) Schematic for the generation of an F_2 population. (**b**) Nucleotide sequence profile of the *Nlrp12* allele in C57BL/6NJ and BALB/cJ mice. (**c, d**) The indicated allele was assessed for all B6N-B6J F2 mice (from Fig. 1f) and cohorts stratified based on their genotype (*B6N/B6N*, *B6N/B6J*, or *B6J/B6J*). N.S. not significant by Mann-Whitney U test.

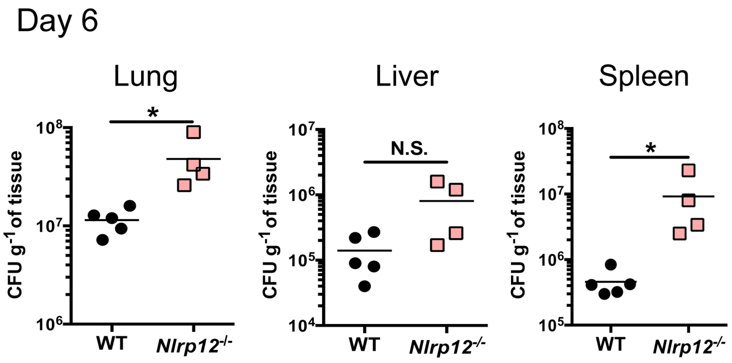

Supplementary Fig. 3. NLRP12 is required for effective control of *F. tularensis* LVS *in vivo*. WT (B6N) and *Nlrp12*$^{-/-}$ mice were infected i.n. with 5 x 10^3 CFU of *F. tularensis* LVS. 6 days post-infection organs were harvested, homogenized and dilutions plated for enumeration of CFU. Data are representative of three independent experiments; * $p < 0.05$ by Mann-Whitney U test.

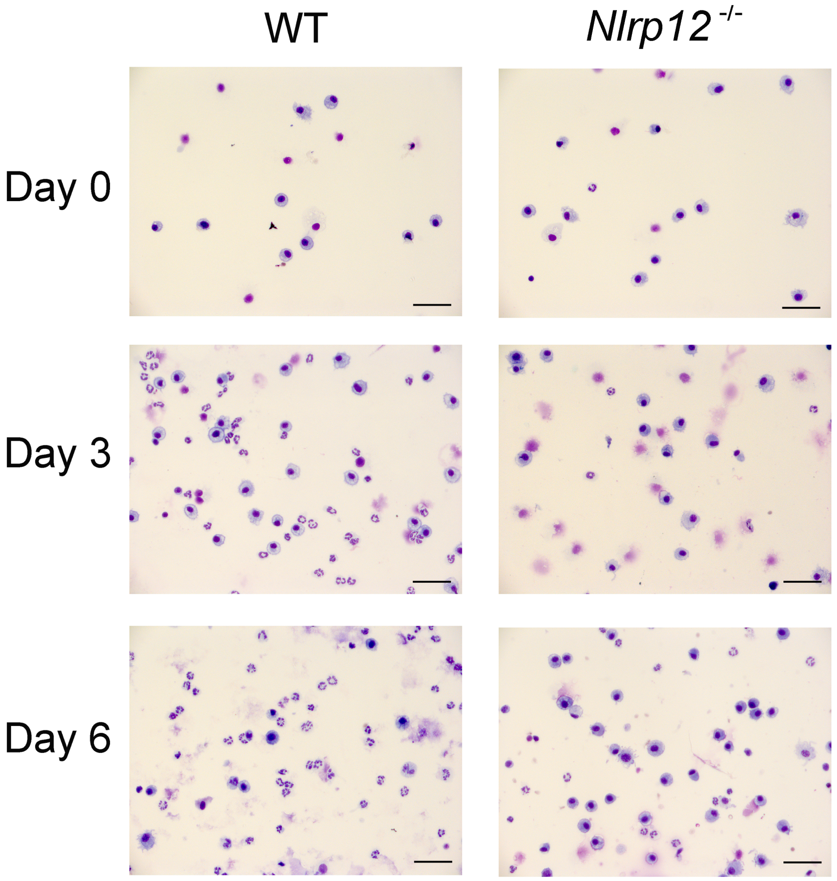

Supplementary Fig. 4. NLRP12 is required for effective neutrophil recruitment to the lungs following infection with *F. tularensis* LVS. WT (B6N) or *Nlrp12*$^{-/-}$ mice were infected i.n. with 5 x 10^3 CFU of *F. tularensis* LVS. At the indicated time post-infection BAL were performed and quantified (Fig. 2c). Representative cytospins are presented. Scale bar = 50 μm.

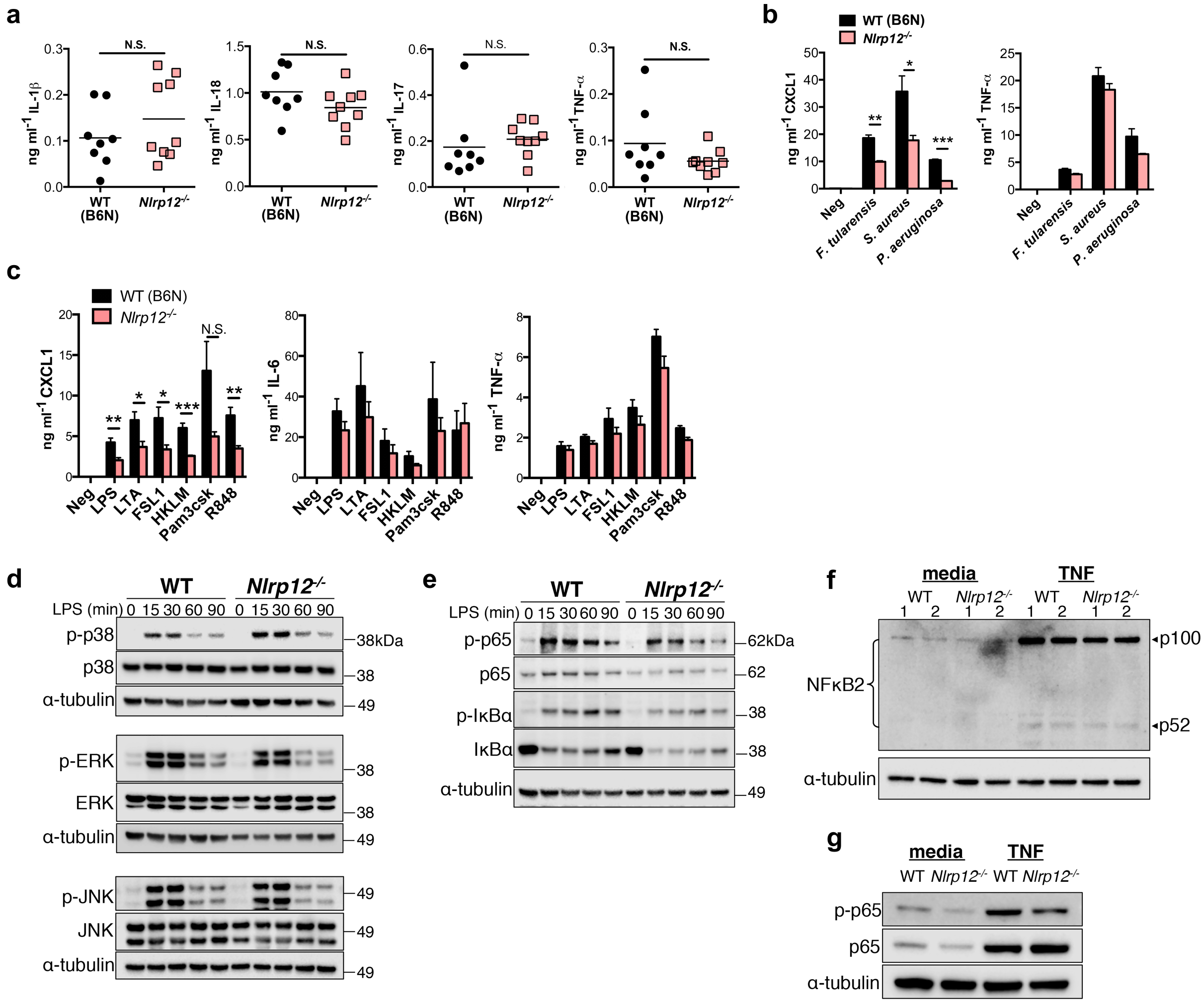

Supplementary Fig. 5. BMDM and BMDC from C57BL/6J and *Nlrp12*$^{-/-}$ mice have defective CXCL1 production. (**a**) WT (B6N) or *Nlrp12*$^{-/-}$ mice were infected i.n. with 5 x 10^3 CFU of *F. tularensis* LVS. 3 days post-infection BAL were performed and cytokine levels were determined by ELISA [n=8, WT; n=9, *Nlrp12*$^{-/-}$]. (**b**) BMDC from WT (B6N) and *Nlrp12*$^{-/-}$ mice were challenged with either *F. tularensis* LVS, *S. aureus,* or *P. aeruginosa*. 8 h later supernatants were collected and secretion of CXCL1 and TNFα was quantified by ELISA. (**c**) BMDM from WT and *Nlrp12*$^{-/-}$ mice were stimulated with the indicated TLR agonist for 8 h or for the indicated amount of time; supernatants were collected and assayed for the indicated cytokines by ELISA. (**d**, **e**) BMDM from WT (B6N) and *Nlrp12*$^{-/-}$ mice were challenged with 50 ng ml^{-1} LPS for the indicated times. Cell lysates were immunoblotted with antibodies against phospho-p38, p38, phosphor-ERK, ERK, phosphor-JNK, JNK, phosphor-p65, p65, phosphor-IκBα, IκBα, and α-tubulin. (**f**, **g**) BMDM from WT (B6N) and *Nlrp12*$^{-/-}$ mice were left unstimulated or challenged with 20 ng ml^{-1} TNFα for 18 h. Cell lysates were immunoblotted with antibodies against p100/p52, phosphor-p65 , p65, and α-tubulin. (**d**-**g**) Data are representative of 2 independent experiments. Pooled data from two (**a**) or three (**b**, **c**) independent experiments are depicted. Data are expressed as the mean ± SEM (**b, c**). * $p < 0.05$, ** $p < 0.01$, *** $p < 0.005$ by Mann-Whitney U test (**a**) or Student's t test (**b, c**).

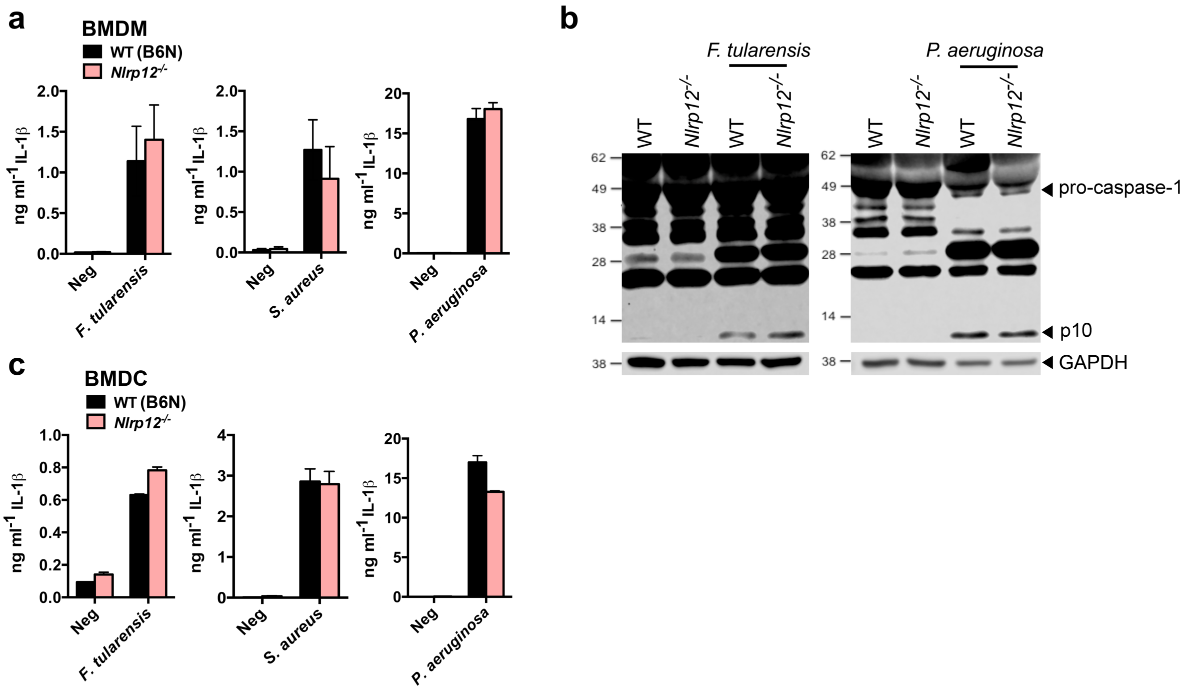

Supplementary Fig. 6. BMDM and BMDC from C57BL/6J and *Nlrp12*$^{-/-}$ mice have intact IL-1β secretion. (**a**-**c**) BMDM (**a**, **b**) or BMDC (**c**) from WT (B6N) and *Nlrp12*$^{-/-}$ mice were challenged with either *F. tularensis* LVS, *S. aureus,* or *P. aeruginosa*. For *F. tularensis* LVS challenge, BMDM and BMDC were primed with LPS (50 ng ml^{-1}) for 4 h prior to challenge. 8 h later supernatants were collected and IL-1β secretion was quantified by ELISA (**a, c**). BMDM lysates were immunoblotted with antibodies against the p10 subunit of caspase-1 and GAPDH (**b**). Pooled data from three independent experiments are depicted (**a**, **c**) or are representative of two independent experiments (**b**). Data are expressed as the mean ± SEM (**a, c**).

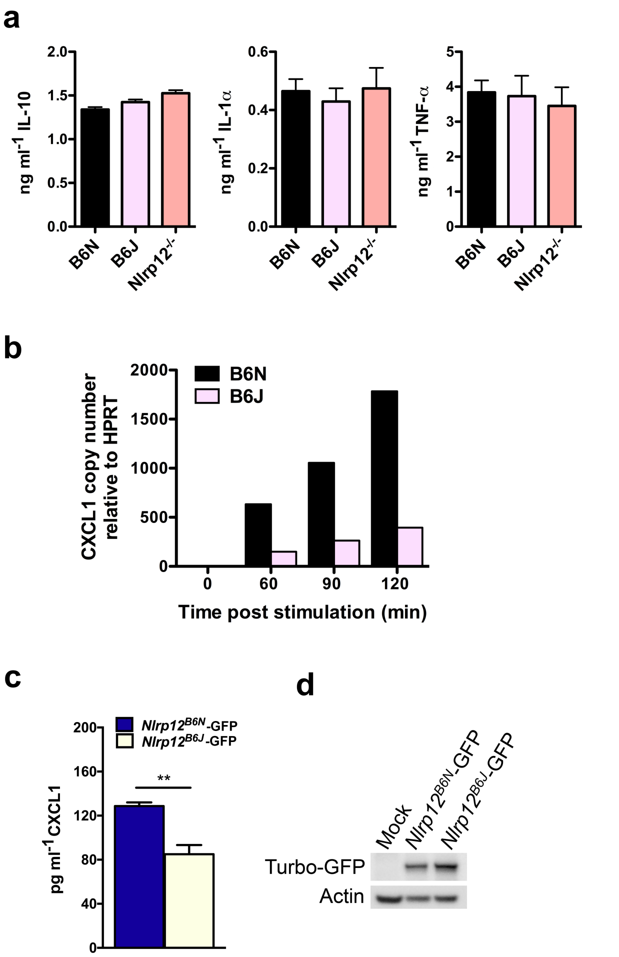

Supplementary Fig. 7. BMDM from C57BL/6J and *Nlrp12*$^{-/-}$ mice have defective CXCL1 production. (**a**) BMDM from WT (B6N), WT (B6J), and *Nlrp12*$^{-/-}$ mice were stimulated with LPS for 8 h; supernatants were collected and assayed for the indicated cytokines by ELISA. (**b**) BMDM from WT (B6N) and WT (B6J) mice were stimulated with LPS for the indicated amount of time; *Cxcl1* expression was quantified by real-time quantitative RT-PCR. (**c**, **d**) HEK-TLR4 cells were transfected with GFP-tagged *Nlrp12*^{B6N} or *Nlrp12*^{B6J}. 24 h after transfection, cells were stimulated for 16 h with 100 ng ml^{-1} LPS; supernatants were collected and assayed for CXCL1 levels by ELISA (**c**) and cell lysates were used for immunoblotted with anti-turboGFP and anti-actin (**d**). Results are representative of three independent experiments (**a**, **c**, **d**). Data are expressed as the mean ± SEM (**a, c**). ** $p < 0.01$ by Student's t test.
